# A Systematic Review of Denture Stomatitis: Predisposing Factors, Clinical Features, Etiology, and Global *Candida* spp. Distribution

**DOI:** 10.3390/jof10050328

**Published:** 2024-04-30

**Authors:** Mirjana Perić, Biljana Miličić, Jovana Kuzmanović Pfićer, Rade Živković, Valentina Arsić Arsenijević

**Affiliations:** 1 Department of Prosthodontics, School of Dental Medicine, University of Belgrade, Rankeova 4, 11000 Belgrade, Serbia; mirjana.peric@stomf.bg.ac.rs (M.P.); rade.zivkovic@stomf.bg.ac.rs (R.Ž.); 2 Department of Medical Statistics and Informatics, School of Dental Medicine, University of Belgrade, Dr. Subotića 1, 11000 Belgrade, Serbia; biljana.milicic@stomf.bg.ac.rs (B.M.); jovana57@yahoo.com (J.K.P.); 3 Medical Mycology Reference Laboratory, Department of Microbiology, Faculty of Medicine, University of Belgrade, Dr. Subotića 4, 11000 Belgrade, Serbia

**Keywords:** denture stomatitis, epidemiology, etiology, predisposing factors, *Candida* spp., global distribution

## Abstract

Denture stomatitis (DS) is a very common disease in wearers of removable complete and partial dentures with a worldwide prevalence in the range of 20–67%. Both industrially developed and impoverished nations are affected by the illness. DS is often associated with ill-fitting dentures or a fungal infection with *Candida* spp. *Candida* is normally found in the oral cavity microbiota, but it can be harmful to the health of elderly people with underlying diseases. Therefore, the purpose of the present study is to offer the most recent information about the epidemiology, etiology, and global distribution of *Candida* species associated with DS through a systematic review. Several databases, including Medline, Web of Science, and Scopus, were used to conduct an extensive search of the literature published in the previous 20 years. The selection of studies was performed by two authors. The extracted data were as follows: author, year of publication, country, sample, frequency of DS, method of diagnosing stomatitis, species of *Candida*, risk factors, and etiology of the disease. The JBI Critical appraisal tools were used to assess the quality of the studies. Eventually, twenty-eight studies were included in the systematic review. Twenty-one studies investigated DS, while seven studies examined *Candida* colonization in patients using removable dentures. The results show that the main causes of DS include the type of dentures, continuous wearing of dentures, and the formation of a *Candida* biofilm, which is facilitated by poor dental hygiene. Additionally, previous studies have pinpointed the significance of the salivary flow, saliva composition, and salivary pH. The findings of the current review indicate that it is crucial to monitor denture wearers for the appearance of DS, especially the patients whose immunity has been impaired due to a systemic condition. Finally, frequent follow-ups should include a clinical examination and microbial swabs of the palatal mucosa and the mucosal surface of the denture.

## 1. Introduction

Denture stomatitis (DS) is a common disease of the oral mucosa. It develops as a consequence of chronic inflammatory processes affecting the areas of the oral mucosa underneath a denture. It is characterized by edema and erythema of the oral mucosa occurring in more severe forms of the disease, along with hyperplastic changes [1]. While it can be accompanied by mild symptoms in the form of a burning or salty sensation in the mouth, DS can also exhibit no symptoms. In the case when the patient does not report any symptoms, the disease is detected during a dental clinical examination. The prevalence of DS among removable denture wearers ranges from 20 to 67% and the condition is mostly detected in elderly patients [2,3,4]. DS is also seen in healthy and younger people who wear orthodontic appliances [5,6].

### 1.1. Etiology

Although the etiology of DS has not been fully determined, it is known that the disease occurrence is influenced by a variety of circumstances. Certain systemic predisposing factors, most notably diabetes mellitus, hypertension, radiation therapy, neoplasms, and HIV infection, as well as conditions involving nutritional deficiency, immunosuppression, immunodeficiency, and hematological disorders, can all impair disease resistance by lowering an individual’s immunity [7,8,9,10,11,12]. Long-term use of corticosteroids and antibiotics can also have an impact on the development of the illness [13]. Local factors include local trauma from an ill-fitting denture, wearing dentures continuously and overnight, dry mouth, denture age, poor denture hygiene, contamination of dentures with a microbial biofilm, carbohydrate-rich diets, acidic salivary pH, and smoking [1,14,15,16,17,18]. In particular, the most prominent factors associated with the development of DS are yeast infections caused by *Candida* spp. and oral mucosal trauma occurring as a result of ill-fitting dentures. Due to the mechanical irritation caused by denture-wearing, inflammation develops in the area of the oral mucosa, which can be an ideal basis for the settlement of microorganisms and the development of an infection.

Although *Candida* is a commensal microorganism that is part of the physiological flora of the oral cavity, it is conditionally pathogenic. Also, a denture itself represents an ideal environment for the retention and growth of microorganisms. This particularly refers to the surface which is in contact with the mucosa. Therefore, wearing a denture is another risk factor that can increase *Candida* colonization, which can consequently lead to the development of a *Candida* biofilm and result in denture stomatitis [4,19,20].

It is known that *C. albicans* is the most abundant species of *Candida.* Yet, in recent years, the prevalence of the so-called non-*albicans Candida* species (*C. glabarta*, *C. krusei*, *C. tropicalis*, *C. parapsilosis*, *C. dubliniensis*) has been increasing.

*Candida* can convert from a commensal yeast to a pathogenic hyphal form under the impact of local and systemic factors. The hyphal form is invasive and can penetrate the host tissue, especially in immunocompromised people. The hyphal form becomes active and begins to generate various hydrolytic enzymes that allow it to stick to host cells and break down their cell walls, thus enabling *Candida* to continue invading the host tissue [1,16,21,22,23].

### 1.2. Clinical Presentation of Denture Stomatitis (DS)

A clinical examination of the mucosa can reveal DS which is usually localized on the palate in upper denture wearers. Owing to saliva’s rinsing and the tongue’s cleaning properties, wearing lower dentures virtually never causes DS on the lower alveolar ridge.

In 1962, Newton divided the clinical signs and symptoms of DS into three groups [24]. The condition was later reclassified by Budtz-Jorgensen and Bertram [25] in accordance with the type of inflammation detected on the mucosa: Type I—the mildest form, which is manifested by localized inflammation, typically as petechiae distributed on the palate; Type II—generalized inflammation, in the form of erythema of variable intensity and localization; and Type III—characterized by papillary hyperplastic inflammation.

### 1.3. Diagnostic Procedure for Denture Stomatitis (DS)

The clinical appearance of a clearly defined area of erythema corresponding to the fitting surface of the denture represents a typical clinical sign of DS and the DS diagnosis can be confirmed by taking denture swabs from the affected surface and conducting a mycological analysis to confirm the presence of *Candida*. A tissue biopsy is not necessary unless the illness also exhibits other questionable symptoms. If a biopsy were to be done, a histological analysis would reveal diminished keratinization and epithelial atrophy, as well as the signs of proliferative or degenerative responses.

Given the worldwide prevalence of DS and the incidence of disease recurrence, identifying DS causal factors is critical for generating effective treatment options. Therefore, the aim of this systematic review is to critically evaluate the available evidence and specifically pinpoint the following aspects in relation to DS:

- Predisposing Factors
- Clinical Features
- Etiology
- Global *Candida* spp. distribution.

In this systematic review, we have determined the presence of *Candida* in patients with DS and classified it according to its species. Moreover, potential factors that may cause the development of DS or a deterioration of the clinical condition have been identified.

## 2. Methods

This systematic review was conducted according to the guidelines of the Preferred Reporting Items for Systematic Reviews and Meta-Analyses—PRISMA Statement 2020 (Supplementary Checklist and Supplementary Checklist Summaries) [26].

### 2.1. Search Strategy

The electronic search was performed in July 2023 in the following databases: MEDLINE (2003 to the present), Web of Science (1980 to the present), and Scopus. The search strategy encompassed a combination of keywords as controlled vocabulary (MeSH—in upper case) and free text terms (in lower case): DENTURE STOMATITIS OR PROSTHETIC STOMATITIS OR ORAL STOMATITIS OR DENTURE INFECTION OR DENTURE-RELATED STOMATITIS AND FUNGAL OR CANDIDA-ASSOCIATED OR CANDIDIASIS OR YEAST SPECIES OR CANDIDA SPECIES AND species AND prevalence OR epidemiology OR rate (Supplementary Search Strategy).

### 2.2. Eligibility Criteria

In terms of the study design, the original articles included in this review represent cross-sectional studies.

#### 2.2.1. Population

The current review included the articles that investigated patients with removable dentures and reported the presence of DS or positive findings regarding *Candida* colonization or infection with *Candida* spp.

#### 2.2.2. Types of Outcomes

The outcome variables that were evaluated in the included studies were: the prevalence of DS, the presence of *Candida*, the detection of different *Candida* spp. and the determination of etiological factors such as age, smoking, denture hygiene, denture age, denture-wearing habits that contribute to DS, and/or *Candida* colonization or infection.

Exclusion criteria were as follows: abstracts, studies older than 20 years, articles including a small study group of participants, studies on animals, and studies on patients who had previously received candidiasis therapy or other treatments for stomatitis.

### 2.3. Study Selection and Data Extraction

The selection of studies was performed by two authors (MP and BM) who first read the titles and abstracts and then considered the full text of the studies that met the inclusion criteria. Data extraction was carried out by two authors (MP and JKP) and independently supervised by another two authors (VAA and BM). The Kappa statistic was 0.75. The extracted data were as follows: author, year of publication, country, sample, frequency of DS, method of diagnosing stomatitis, *Candida* species, risk factors, and etiology of the disease.

### 2.4. Methodological Quality Assessment

The JBI Critical appraisal tools (Checklist for Analytical Cross-Sectional Studies) were used to assess the quality of the eligible studies and possible risk of bias [27]. This tool consists of 8 domains related to: clear inclusion criteria, detailed setting description, valid/reliable exposure, objective/standard measurement criteria, confounding factor identification, strategies to deal with confounding factors, valid/reliable outcome measurement, and appropriate statistical analysis.

## 3. Results

### 3.1. Study Characteristics

**Figure 1:**
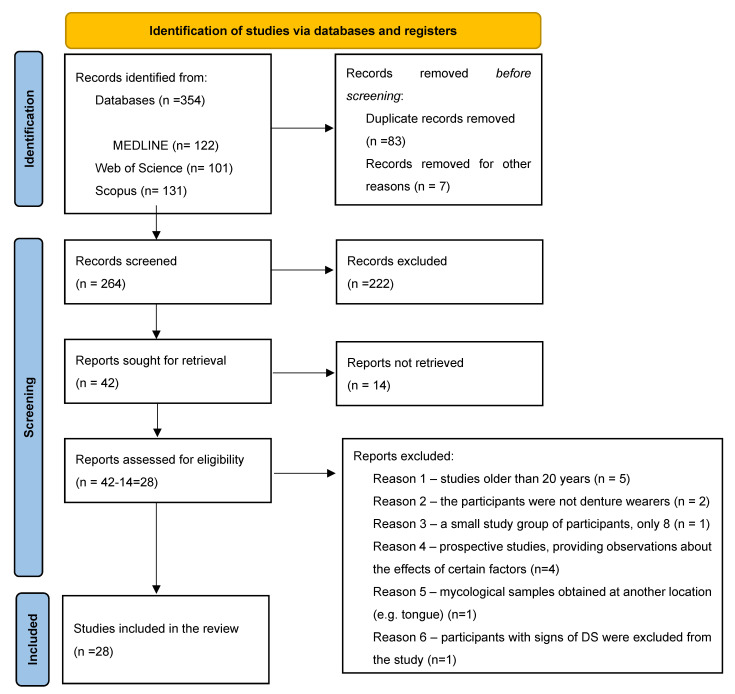
Flow chart diagram.

The number of studies identified through the selection process is shown in Figure 1.

Twenty-eight studies were included in the systematic review [2,18,21,22,28,29,30,31,32,33,34,35,36,37,38,39,40,41,42,43,44,45,46,47,48,49,50]. Twenty-one studies investigated DS [2,18,21,22,28,29,30,31,32,33,34,35,36,37,38,39,40,41,42,43,44], while seven studies [45,46,47,48,49,50,51] examined *Candida* colonization in patients using removable dentures.

In terms of the continent where the studies were carried out, the majority of them were conducted in Asia 44.4% (n = 12), followed by North and South America 33.3% (n = 9). In other words, about two-thirds of the included studies were conducted on these two continents (Table 1 and Table 2).

The total number of included participants was 4243 and they were divided into two groups: (i) the patients with DS (3789 patients); and (ii) the patients with *Candida* colonization (454 patients). Table 1 and Table 2 display the summaries of the included studies. Out of a total of 3789 examined patients, DS was confirmed in 2237 (59%) patients in the first group. Regarding the latter group with *Candida* colonization, the yeast was found in a total of 337 (74.2%) out of 454 participants.

Most patient samples were collected in Poland (24.2%; n = 920), South Africa (7.8%; n = 294), and Yemen (7.6%; n = 288), while the lowest numbers of samples were obtained in Brazil (0.79%; n = 30) and the United States (0.84%; n = 32).

The majority of investigations [21,29,34,35,39,41,42] used swabbing methods to collect microbiological material from the diseased mucosa of the palate and the denture-fitting surface. In some studies, only a swab was obtained—either from the palatal mucosa [30,33,43] or from a denture-fitting surface [32,37,38,40]. In the studies of Abaci et al. [2] and Pereira et al. [31], a swab and an unstimulated saliva sample were used. In the study of Perić et al. [44], three different methods were used—a swab, oral rinse, and denture sonication. Finally, oral rinse sampling [46,48,50,51] and oral swabs [45,47,48,49] were most frequently used in the study group in which *Candida* colonization occurred.

According to ten different studies [2,22,30,31,32,34,37,38,39,42], an important etiological factor for the development of DS is the presence of *Candida* in the oral cavity and on the dentures. However, twelve investigations [18,21,28,29,33,34,36,37,38,39,43,44] concluded that the current condition of the dentures, their continuous use, and their presence in the oral cavity all have an effect on the development of DS. According to the following studies [28,36,37,43,44], the age of the denture is an important factor in the development of DS. Furthermore, three studies [2,33,39] identified poor oral hygiene and denture hygiene as the dominant factors in the occurrence of the disease. Numerous studies demonstrated that saliva, i.e., its volume and pH, might have an impact [33,43,51] on DS development. While five studies [18,28,37,42,44] indicated the patient’s age as a risk factor for the occurrence of DS, and four studies [28,37,40,42] highlighted the patient’s gender as a predisposing factor for its development. The following studies demonstrated that certain patient behaviors, such as smoking, can have a significant impact on the onset of DS: Barbeu et al. [21] and Perić et al. [44]. Four investigations emphasized the influence of systemic diseases (e.g., diabetes mellitus and hypertension) on the emergence of DS [30,32,33,51].

A number of studies identified the predominant etiological factors of DS in patients with *Candida* spp. colonization: use of a denture—four studies [45,46,49,51], poor oral hygiene—three studies [45,47,48], xerostomia and low pH of saliva—four studies [46,48,50,51], and the presence of *Candida* in the oral rinse—two studies [48,50].

### 3.2. Quality Assessment

The results of the studies evaluated for quality assessment are presented in Table 3 and Table 4. In Table 3, the results show that all studies met the population selection and inclusion criteria, except for one investigation in which this segment remained unclear [30]. In one study, the validity of exposure measurement was unclear [40]. In three studies, it was uncertain how DS was diagnosed [30,40,42], while one study did not provide any information about the diagnosis [37]. In four studies, the identification of confounding factors was vaguely described [30,31,40,41]. Eight studies, out of 21 in total, dealt with the influence of confounding factors using regression models [21,28,32,37,38,39,43,44]. Lastly, three studies did not adequately describe the statistics [29,33,40].

In Table 4, the results of the study quality assessment show that in three studies, the confounding factors were not clearly identified [46,49,50]. Only one study analyzed the influence of confounding factors through a regression model [51]. Two studies did not clearly report statistics [45,49].

## 4. Discussion

### 4.1. Epidemiology

The studies examining DS are presented in Table 1. Since individuals wearing complete dentures are susceptible to DS, several studies further reported the prevalence rates of DS solely among these individuals. The prevalence of DS ranged from 11% to 70%. In two studies by Dagestan et al. [34] and Barbeau et al. [21], the DS prevalence in denture wearers was 70%. Due to the small number of examined denture wearers (about 70), the data provided may need to be interpreted with caution. The majority of investigations showed a 30–40% prevalence rate of DS: Adam et al. [28], Sanita et al. [32], Kossioni et al. [18], Perić et al. [44], and Altarawneh et al. [22] (34.69%, 38.09%, 32.62%, 32.88%, and 46.77%, respectively).

Various reports found differences in the occurrence of DS in relation to gender. Some studies claimed that men are more likely to be affected [40,49,52], while other studies stated that DS is more prevalent among women [42]. The elderly are most commonly affected, with people over 50 years of age having the highest chance of developing this condition [28,38,42,44,49,52].

### 4.2. Clinical Features

Notably, distinct but quite comparable scores, proposed by Newton [24] and Budtz-Jorgensen and Bertram [25], are used to describe DS clinically (scores derived from either scale can be interpolated to the other). The clinical signs of the Newtonian Type I and Type II were the most common. Among all analyzed studies, only the study by Kaomongkolgit et al. [52] found that Type III of denture stomatitis (papillary hyperplasia) was the most common clinical type of the disease.

Nevertheless, some publications failed to mention that they used the given scores and to display the illness distribution based on the severity of the clinical picture.

### 4.3. Etiology of DS and Global Candida Distribution

From an epidemiological standpoint, it is possible to state that a spectrum of similar species of the genus *Candida* causing DS has been isolated and identified worldwide (Table 1). In the presented studies, *C. albicans* was the most frequently reported species. It was isolated from diseased tissues either alone or in a combination with other so-called non-*albicans Candida* species, including *C. tropicalis*, *C. parapsilosis*, *C. glabrata*, *C. krusei*, *C. dubliniensis*. Three species of *Candida* have been associated with the development of DS in North America: *C. albicans*, *C. glabrata*, and *C. tropicalis.* The four studies carried out in Brazil, which accounts for the greatest number of studies conducted in South America, have indicated the following species as causal agents of DS: *C. albicans*, *C. glabrata*, *C. tropicalis*, *C. krusei*, *C. famata*, *C. dubliniensis*, *C. guilliermondii*, and *C. lusitaniae*. In Asia, the following *Candida* species have been isolated from patients with DS: *C. albicans*, *C. glabrata*, *C. krusei*, *C. tropicalis*, *C. pseudotropicalis*, *C. kefyr*, *C. famata*, *C. sphaerica*, *C. guilliermondii*, *C. lipolytica*, *C. boidini*, and *C. norvegensis*. Several *Candida* species, including *C. albicans*, *C. glabrata*, *C. dubliniensis*, *C. tropicalalis*, and C. *krusei*, have been isolated in patients from Europe.

In recent years, *Candida* spp. linked to DS have shifted from *C. albicans* to non-*albicans Candida* species. It should be noted that an increased use of antifungals worldwide has contributed to this phenomenon.

The second most prevalent species after *C. albicans* is *C. glabrata* which is frequently isolated from the surfaces of acrylic dentures and the palatal mucosa. It is currently thought that the rise in *C. glabrata* infections has been caused by an increased use of immunosuppressive medications. Additionally, there is a clear propensity for many species of *Candida* to simultaneously colonize the mucosa both in healthy individuals and in those with underlying conditions.

According to numerous studies, several species of *Candida* can exhibit specific virulence properties under certain conditions, which enables colonization and infection of the host. *Candida* can adhere to acrylic resin dentures directly or through the plaque layer. This is the initial step that can cause the appearance of an infectious process. Without this adhesion, the germs would be expelled from the oral cavity when food or saliva is swallowed [53]. *Candida* adheres to acrylic resin due to various characteristics, including the surface charge, free energy, hydrophobicity, and roughness. All of these properties affect how the microbes initially behave.

It should be mentioned that earlier reviews and studies established that the etiology of DS is multifaceted. Several studies also indicated that certain risk factors may be linked to the patient’s use of a denture. The mere presence of a denture in the oral cavity alters its physiological parameters. Many studies have found that the presence of a denture is a risk factor for the colonization of *Candida* spp. and development of DS [46,51].

Patients with brand-new conventional complete dentures were monitored by Mousa et al. [54]. Their objective was to determine whether new dentures have an impact on *Candida* colonization and DS development. Mycological samples from the participants’ palatal mucosa and the fitting surfaces of the corresponding dentures were taken on the day of the denture placement and during the follow-up period. On the day of denture insertion, but prior to placing the denture, 58.7% of patients already had colonies of various *Candida* species with *C. albicans* being the most predominant species. In contrast to the first month when no subjects received a DS diagnosis, DS was confirmed in a total of 17.2% of study participants during the second month. All of them were assigned Type I according to the Newton classification.

Zomordian et al. [55] included 114 participants with complete dentures in their study. Following an examination of the oral cavity, DS was detected in 53 (46.5%) of the 100/114 denture users with yeast colonization (87.7%). The counts of ≥50 CFU/mL of *Candida* colonies met the criteria for defining a *Candida*-associated DS infection.

The only variable that significantly affected the level of infection was the duration of wearing dentures—DS was found in 25% of those who had been wearing dentures for less than a year; in 53.3% of patients who had been wearing dentures for one to five years; and in 84.2% of patients who had been wearing dentures for more than five years.

It should be pointed out that the consequences of colonization with *Candida* species should not be underestimated. Although it can usually be found in 30% to 70% of individuals, often no clinical signs may be detected. It should be kept in mind that this microorganism can become pathogenic in the case of a quantitative growth as high CFU/mL counts coincide with increased frequencies of DS.

Recent research has shown that DS can emerge more rapidly than previously thought and is frequently linked to mixed *Candida* spp. In addition, improperly fitting dentures, poor denture hygiene, and sleeping with dentures all have profound effects on the onset of DS and *Candida* colonization.

What primarily characterizes *Candida*-related DS is a large-scale isolation of *Candida* spp. (>50 CFU/mL). However, the definitions of infection and colonization can differ. For example, different thresholds can be used when it comes to the amount of CFU/mL that indicates an infection.

DS etiology involves a lack of adequate denture cleanliness, inflammation, and trauma that appears as a result of wearing improper dentures, increasing denture age, and continuous denture usage. Allergy to denture material has also been identified as a possible cause in the etiology of DS. In particular, poor oral hygiene has been identified as a major risk factor for the development of DS in this scenario. For instance, some studies have found that wearing dentures while sleeping is significantly associated with poor dental hygiene. Furthermore, poor oral hygiene is related to increased *Candida* colonization of the oral mucosa and dentures [2,31,33,34,37,39,48]. The constant use of dentures, in particular, stands out as a major component in the development of DS [16,18,21,28,33,36,37,38,52,54]. Wearing dentures overnight, when saliva output in the mouth cavity is limited, is what creates ideal conditions for the disease to develop.

Some studies have also highlighted the importance of denture structure and material in the development of DS. The propensity of the acrylic base of the denture (polydimethyl methacrylate) towards the adhesion of *Candida* spp. and the formation of biofilms is particularly noteworthy in relation to the base of a denture made of a metal Co Cr Mo alloy. The surface of the acrylic base of a denture is slightly porous and represents an ideal environment for retaining a denture biofilm (plaque). The significance of the overdenture retention mechanism is highlighted in the study by Kilić et al. [35] which examined CFU values of Candida species with regard to the use of bar or locator attachments. The study concluded that, in comparison with the locator system, a *Candida* biofilm is more likely to adhere to the bar system. It must be noted that both retention techniques need the highest levels of cleanliness and control.

The age of the denture [28,36,37,55] is another crucial factor. Certain changes in the acrylic material occur over time, thus increasing the porosity and making cleaning the denture more difficult. As a result, denture plaque retention is aided by these factors. In accordance with previous observations, we can state that DS in denture wearers increases with a prolonged use of dentures.

Last but not least, a person may be predisposed to developing this infection if they already have other diseases or disorders that may require treatment. These include diabetes [9,15,33,56], hypertension [9,15,33], immunosuppressive therapy [57], extended systemic antibiotic treatment [58], and high carbohydrate ingestion [9,15].

Salivary pH has also been investigated as a potential risk factor for the onset of DS. *Candida* infection is facilitated by the oral cavity’s acidic environment. In patients with DS, Chope et al. [15] found a salivary pH value of 5.1 and a similar value of 5.2 was reported by Baena-Monroy et al. [9]. According to Pires-Goncalves et al. [46], a pH value of 6.5 is the lowest limit, i.e., below this value there is a higher likelihood of *Candida* spp. colonization.

Al-Dwairi et al. [59] reported that a total of 70% of subjects with Type III DS were heavy smokers (more than 15 cigarettes/day). On the other hand, Perić et al. [44] pointed out that the status of an ex-smoker can be a factor in the development of DS. Although the exact explanation of smokers’ increased risk for developing DS is unknown, it is believed that localized epithelial changes are caused by aromatic compounds in the smoke [60]. Unexpectedly, a study by Martori et al. [61] found a significant association between the occurrence of DS and never-smoking individuals.

Due to the ability of glucose to stimulate the growth of *Candida* spp. and increase the adherence of yeast within the dental plaque, denture wearers with a high sugar intake are also at an increased risk of developing DS. Poor denture cleanliness and sugar consumption may both have a role in the development of DS [60]. Therefore, opting for effective treatment modalities is of paramount importance. Patients who wear dentures are mostly elderly people who have several comorbidities that make them susceptible to DS.; This is to say that prevention, treatment, and education on how to prevent DS are all of vital importance. As part of patient education, these patients should be provided with clear guidance on maintaining proper denture hygiene, including advice on how to adequately brush their dentures. So far, encouraging results have been reported regarding both natural and synthetic antimicrobial agents incorporated into brushes and toothpastes [62,63,64]. Moreover, it is essential to ensure disinfection of the denture using agents for removing prosthetic plaque such as: chlorhexidine gluconate 2%, sodium hypochlorite 0.5% or 1%, alkaline peroxide, or effervescent tablets [65,66,67,68]. It might be desirable for patients with general diseases to use natural products preventively. They can be used for rinsing the mouth or for immersing the denture completely, which represents another preventive measure. This should be done from the moment of denture placement. In some cases, it is necessary to apply antifungal agents such as: amphotericin B, miconazole 2% gel applied locally or fluconazole 50 mg applied systemically in the case of advanced DS [65,69]. In particular, the significance of follow-up examinations should be emphasized since they enable the doctor to evaluate the degree of denture hygiene and motivate the patients [68,70], but also to obtain mycological findings of different *Candida* spp. [44].

The limitation of this systematic review is the inconsistency of the results reported in the included studies. This can be explained by deficiencies in the data presented in the studies since all included studies have been assessed for quality and risk of bias.

## 5. Conclusions

Finally, we can state that even in healthy denture users, the presence of *Candida* should be regarded as a risk factor for DS. The risk of DS development increases with prolonged denture usage.

Patient monitoring is essential, and it should comprise frequent examinations of the oral cavity by taking swabs from the palatal mucosa and the denture’s mucosal surface for mycological investigations. We especially advise swabbing the palatal mucosa to check for a *Candida* spp. infection prior to placing a new denture. If there is *Candida* colonization, routine examinations should be carried out.

In order to lower the risk of DS, patients should be provided with clear instructions on how to properly care for the denture and how to maintain its hygiene. Also, a recommendation not to wear the denture at night is mandatory.

## Figures and Tables

**Table 1:** Descriptive characteristics of included studies, i.e., individuals with denture stomatitis by country, clinical symptoms, *Candida* species (*C*.), and risk factors.

References, Year	Country (Sample Size)	Identified Fungal Species	Predisposing Factors, Etiology	SCOR
A F R I C A				
Adam, Z.A. et al., 2021 [28]	South Africa (294 patients 102 with DS *)	N/A **	Age of denture Age of subjects Overnight denture wearing	N/A
A M E R I C A				
Altarawneh, S. et al., 2013 [22]	USA (32 patients/15 with DS)	*C. albicans* 71.4% *Non-albicans* 28.6%	Presence of *Candida* in denture and saliva samples	Type II—53.3% Type III—46.6%
Barbeu, J. et al, 2003 [21]	Canada (68 patients/48 with DS)	*C. albicans*, *C. glabrata*, *C. tropicalis*	Overnight denture wearing Smoking	Type I—12.5% Type II—35.4% Type III—52%
Gauch L.M.R. et al., 2018 [29]	Brazil (36 patients with DS)	*C. albicans*, *C. famata*, *C. tropicalis*, *C. parapsilosis*	Unsatisfactory denture condition	Type I—50% Type II—33% Type III—17%
Motta-Silva, A. C. et al., 2010 [30]	Brazil (247 patients/28 patients with DS and DM ***)	*C. albicans*, *C. glabrata*, *C. tropicalis. C. krusei*, *C. famata*	Patient with DM had: a greater diversity of *Candida* spp. proteinase production by *C. albicans*	N/A
Pereira, C. A. et al., 2013 [31]	Brazil (100 patients with denture/50 patients with DS/50 patients without DS)	*C. albicans*, *C. glabrata*, *C. tropicalis*, *C. dubliniensis*, *C. guilliermondii*, *C. krusei*, *C. lusitaniae*	Counts of microorganisms on the denture-fitting surface	Type I—42% Type II—48% Type III—10%
Sanita, P. V. et al., 2011 [32]	Brazil (210 patients/80 patients with DS/40 patients with DS + DM)	*C. albicans*, *C. glabrata*, *C. tropicalis*	Prevalence of *Candida* spp. was similar between the groups with DS + DM and DS Prevalence of *C. tropicalis* increased with the higher degree of DS	DS Without DM Type I—20% Type II—61% Type III—19% DS with DM Type I—38% Type II—53% Type III—10%
Qiu, J. et al., 2023 [33]	Brazil (30 patients with DS)	*C. albicans*, *C. glabrata*, *C. tropicalis*, *C. parapsilosis*, *C. dubliniensis*	Hyposalivation Overnight denture wearing Inadequate denture cleaning Poor denture adaptation Hypertension, DM	Type I—56.7% Type II—23.3% Type III—20%
A S I A				
Abaci, O. et al., 2010 [2]	Turkey (110 patients)	*C. albicans*, *C.glabrata*, *C.krusei*, *C.kefyr*, *C.famata*, *C.sphaerica*	Denture hygiene ˃400 CFU/ml	Type I—58.8% Type II—41.2%
Dagistan, S. et al., 2009 [34]	Turkey (70 patients with dentures/49 patients with DS)	*C. albicans*, *C. krusei*, *C. pseudotropicalis*, *C. guilliermondii*, *C. lipolytica*, *C. boidini*	*Candida* spp. infection	Type I—65% Type II—21% Type III—14%
Kilic, K. et al., 2014 [35]	Turkey (37 patients)	*C. albicans*, *C. glabrata*, *C. kefyr*, *C. norvegensis*	DS developed in all patients using bar-retained overdentures, it developed in only 71.4% of patients using locator-retained overdentures	N/A
Navabi, N. et al., 2013 [36]	Iran (70 patients/43 with DS)	N/A	Denture age Overnight denture wearing The practitioner manufacturing denture	Type I—62.79% Type III—4.65%
Al Kebsi, A.M. et al., 2018 [37]	Yemen (288 patients with DS)	N/A	Poor denture fitting Higher denture age Inadequate oral/denture hygiene Overnight denture wearing	N/A
Al-Sanabani, N.F. et al., 2018. [38]	Yemen (288 patients with DS)	*C. albicans*, *C. glabrata*,	Gender (male) Older age (65+ years)	Type I—66.7% Type II—33.3% Type III—0
Aoun, G. et al., 2016 [39]	Lebanon (60 patients with DS)	N/A	Continuous use of the denture, denture hygiene, denture colonization by *Candida*	N/A
Bhat, V. et al., 2013 [40]	India (55 patients/27 patients with DS)	*C. albicans*, *C. tropicalis*, *C. glabrata*	Gender (male)	N/A
E U R O P E				
Calcattera, R. et al., 2013 [41]	Italy (190 patients/126 patients with DS)	*C. albicans*, *C. glabrata*, *C. dubliniensis*, *C. tropicalis*, *C. krusei*	N/A	N/A
Kossioni, A.E. 2009 [18]	Greece (106 patients/42 patients with DS)	N/A	Continuous denture use, increased duration of denture wearing, and the consequent alterations in denture characteristics (e. g. poor retention, reduced occlusal vertical dimension) predisposed to the condition.	Type I—17% Type II—16% Type III—6.6%
Loster, J.E. et al., 2016 [42]	Poland (920 patients with dentures/542 with oral yeast infection)	*C. albicans*, *C. glabrata*, *C.*, *C. tropicalis*	Gender and age of subjects (˃50 years higher proclivity for oral *Candida* infections)	N/A
Čanković, M. et al., 2017 [43]	Serbia 150 patients with dentures/50 patients with DS)	N/A	overnight denture wearing denture age acidic pH value of saliva	Type I—48% Type II—24% Type III—28%
Perić, M. et al., 2018 [44]	Serbia 250 patients with dentures/82 patients with DS	*C. albicans*, *C. glabrata*, *C.*, *C. tropicalis*, *C. krusei*	Age of subjects Increased age of the mandibular denture Ex-smokers	Type I—25.8% Type II—59.8% Type III—14.4%

* DS, denture stomatitis, ** N/A, not available, *** DM, diabetes mellitus.

**Table 2:** Descriptive characteristics of included studies, i.e., individuals with denture colonization by country, clinical symptoms, *Candida* species (*C*.), and risk factors.

References, Year	Country (Sample Size)	Sampling Method	Identified Fungal Species	Predisposing Factors, Etiology
A M E R I C A				
da Silva N. P. et al., 2015 [45]	Brazil (44 patients with dentures/34 with *Candida* colonization)	Swab of the mucosa of the palate	*C. albicans*, *C. tropicalis*, *C. krusei*	Poor oral hygiene Poor denture adaptation
Pires-Goncalves, R. H. et al., 2007 [46]	Brazil (133 with dentures/91 patients with *Candida* colonization)	Oral rinse	*C. albicans C. parapsilosis*, *C. tropicalis*, C. glabrata C. krusei *C. rugosa*	Use of denture Saliva pH < 6.5
A S I A				
Khaje Hosseini, S. K. et al., 2014 [47]	Iran (100 patients with dentures/42 *Candida* colonization of the denture)	Swab of the mucosal surface of the denture base	N/A *	Poor oral hygiene Smoking habit Systemic disease
Ozaki, K. et al., 2022 [48]	Japan (32 patients/19 *Candida* colonization new denture after 6 months)	Swab of the mucosal surface of the denture base	*C. albicans*, *C. glabrata*, *C. parapsilosis.*	Xerostomia, denture cleaning habits, *Candida* carriage in the palatal mucosa, and *Candida* carriage in the oral rinse
Samnieng, P. et al., 2017 [51]	Thailand (102 patients with DM **/36 with dentures/26 *Candida* colonization)	Oral rinse	*C. albicans*, *C. glabrata*, *C. tropicalis*, *C. krusei*	Salivary pH and the use of a denture
Prakash B. et al., 2015 [49]	India (100 patients/50 without dentures/50 with dentures, all with *Candida* colonization)	Swab of the mucosal surface of the denture base	*C. albicans*, *C. tropicalis*, *C. dubliensis*, *C. glabrata.*	Age, Gender (male), Use of denture
E U R O P E				
Kinkela Devčić, M. K. et al., 2021 [50]	Croatia 120 patients/80 with dentures/75 patients with *Candida* colonization	Oral rinse	*C.albicans*, *C. glabrata*, *C.tropicalis*, *C.krusei*	˃600 CFU/mL The lowest saliva flow rate in patients with acrylic dentures

* N/A, not available, ** DM, diabetes mellitus.

**Table 3:** Quality assessment of included studies investigating individuals with denture stomatitis (JBI Cross-Sectional Studies Checklist).

Studies	Clear Inclusion Criteria	Detailed Setting Description	Valid/Reliable Exposure	Objective/ Standard Measurement Criteria	Confounding Factor Identification	Dealing Strategies for Confounding Factors	Valid Reliable Outcome Measurement	Appropriate Statistical Analysis	Quality Score
Adam, Z.A., 2021 [28]	yes	Yes	unclear	yes	yes	Yes	yes	yes	7/8
Altarawneh, S., et al., 2013 [22]	yes	Yes	yes	yes	yes	No	yes	yes	7/8
Barbeu, J. et al., 2003 [21]	yes	Yes	yes	yes	yes	Yes	yes	yes	8/8
Gauch, L.M.R. et al., 2018 [29]	yes	Yes	yes	yes	yes	No	yes	unclear	6/8
Motta-Silva, A.C. et al., 2010 [30]	yes	unclear	yes	unclear	unclear	No	yes	yes	4/8
Pereira, C.A. et al., 2013 [31]	yes	Yes	yes	yes	unclear	No	yes	yes	6/8
Sanita, P.V. et al., 2011 [32]	yes	Yes	yes	yes	yes	Yes	yes	yes	8/8
Qiu, J. et al., 2023 [33]	yes	Yes	yes	yes	yes	No	yes	unclear	6/8
Abaci, O., et al., 2010 [2]	yes	Yes	yes	yes	yes	No	yes	yes	7/8
Dagistan, S. et al., 2009 [34]	yes	Yes	yes	yes	yes	No	yes	yes	7/8
Kilic, K. et al., 2014 [35]	yes	Yes	yes	yes	yes	No	yes	yes	7/8
Navabi, N., et al., 2013 [36]	yes	Yes	yes	yes	yes	No	yes	yes	7/8
Al Kebsi, A.M., et al., 2018 [37]	yes	Yes	yes	no	yes	Yes	yes	yes	7/8
Al-Sanabani, et al., 2018 [38]	yes	Yes	yes	yes	yes	Yes	yes	yes	8/8
Aoun, G., et al., 2016 [39]	yes	Yes	yes	yes	yes	Yes	yes	yes	8/8
Bhat, V. et al., 2013 [40]	yes	Yes	unclear	unclear	unclear	No	yes	unclear	3/8
Calcattera, R. et al., 2013 [41]	yes	Yes	yes	yes	unclear	No	yes	yes	6/8
Kossioni, A.E. 2009 [18]	yes	Yes	yes	yes	yes	No	yes	yes	7/8
Loster, J.E. et al., 2016 [42]	yes	Yes	yes	unclear	yes	No	yes	yes	6/8
Čankovic, M. et al., 2017 [43]	yes	Yes	yes	yes	yes	Yes	yes	yes	8/8
Perić, M. et al., 2018 [44]	yes	Yes	yes	yes	yes	Yes	yes	yes	8/8

**Table 4:** Quality assessment of included studies investigating individuals with denture colonization (JBI Cross-Sectional Studies Checklist).

Studies	Clear Inclusion Criteria	Detailed Setting Description	Valid/Reliable Exposure	Objective/ Standard Measurement Criteria	Confounding Factor Identification	Dealing Strategies for Confounding Factors	Valid Reliable Outcome Measurement	Appropriate Statistical Analysis	Quality Score
da Silva N.P. et al., 2015 [45]	yes	Yes	yes	yes	yes	No	yes	unclear	6/8
Pires-Goncalves, R. H. et al., 2007 [46]	yes	Yes	yes	yes	unclear	No	yes	yes	6/8
Khaje Hosseini, S. K. et al., 2014 [47]	yes	Yes	yes	yes	yes	No	yes	yes	7/8
Ozaki, K. et al., 2022 [48]	yes	Yes	yes	yes	yes	No	yes	yes	7/8
Samnieng, P. et al., 2017 [51]	yes	Yes	yes	yes	yes	Yes	yes	yes	8/8
Parakash, B. et al., 2015 [49]	yes	Yes	yes	yes	unclear	No	yes	unclear	5/8
Kinkela Devcic, M.K. et al., 2021 [50]	yes	Yes	yes	yes	unclear	No	yes	yes	6/8

## Data Availability

No new data were created or analyzed in this study. Data sharing is not applicable to this article.

## Supplementary Materials

The following supporting information can be downloaded at: https://www.mdpi.com/article/10.3390/jof10050328/s1.
